# Task-Dependent Modulation of Medial Geniculate Body Is Behaviorally Relevant for Speech Recognition

**DOI:** 10.1016/j.cub.2008.10.052

**Published:** 2008-12-09

**Authors:** Katharina von Kriegstein, Roy D. Patterson, T.D. Griffiths

**Affiliations:** 1 Wellcome Trust Centre for Neuroimaging, University College London, Queen Square, London WC1N 3BG, UK; 2 Medical School, University of Newcastle upon-Tyne, Framlington Place, Newcastle-upon-Tyne NE2 4HH, UK; 3 Centre for the Neural Basis of Hearing, University of Cambridge, Downing Street, Cambridge CB2 3EG, UK

**Keywords:** SYSNEURO

## Abstract

Recent work has shown that responses in first-order sensory thalamic nuclei are modulated by cortical areas [1–5]. However, the functional role of such corticothalamic modulation and its relevance for human perception is still unclear. Here, we show in two functional magnetic resonance imaging (fMRI) studies that the neuronal response in the first-order auditory thalamus, the medial geniculate body (MGB), is increased when rapidly varying spectrotemporal features of speech sounds are processed, as compared to processing slowly varying spectrotemporal features of the same sounds. The strength of this task-dependent modulation is positively correlated with the speech recognition scores of individual subjects. These results show that task-dependent modulation of the MGB serves the processing of specific features of speech sounds and is behaviorally relevant for speech recognition. Our findings suggest that the first-order auditory thalamus is not simply a nonspecific gatekeeper controlled by attention [6]. Together with studies in nonhuman mammals [4, 5], our findings imply a mechanism in which the first-order auditory thalamus, possibly by corticothalamic modulation, reacts adaptively to features of sensory input.

## Results and Discussion

The anatomical and physiological similarity of first-order thalamic nuclei in different sensory modalities has led to the assumption that they perform general functions in the processing of sensory information [7–11]. But the functions themselves remain to be specified. Recent functional magnetic resonance imaging (fMRI) has shown that the first-order sensory thalamus in humans is modulated by top-down attention [6]. This finding supports a picture of the first-order sensory thalamus as an early, but relatively nonspecific gatekeeper for sensory information [6, 12]. However, electrophysiological studies in nonhuman mammals suggest a more specific role for first-order thalamic nuclei [1–5, 13, 14]. It has been proposed that thalamic receptive fields are tuned by cortical areas to optimize processing of the dynamically varying sensory input [4, 14–16]. For example, in the auditory system, corticofugal connections are thought to mediate dynamic changes in the response properties of first-order thalamic receptive fields for species-specific communication sounds [5], either directly or through the thalamic reticular nucleus (TRN). The first study reported in this paper tested whether modulation by nonspecific attention is sufficient to explain top-down modulation of the medial geniculate body (MGB) in humans (experiment 1). Note that “top-down” here refers to all types of effects (e.g., attentional, task dependent) that are not elicited directly by stimulus input (which would be “bottom-up”) [17, 18]. However, we do not necessarily imply a specific neuronal mechanism (e.g., direct cortico-thalamic modulation).

**Figure 1:**
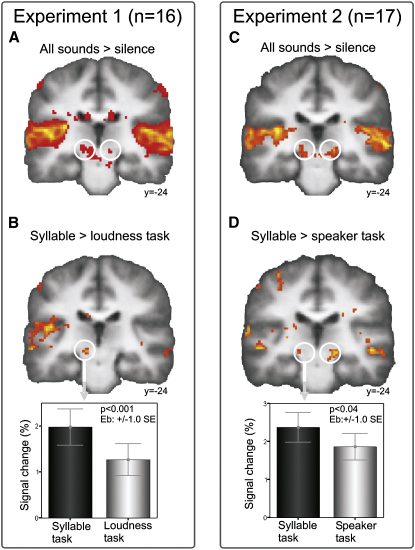
Categorical Analysis Experiment 1 (A and B) experiment 2 (C and D). Group statistical parametric maps are rendered on coronal sections of the group-mean normalized, structural MRI volume. Plots show parameter estimates extracted from the left sensory auditory thalamus, the medial geniculate body (MGB) for experimental conditions contrasted against the silent baseline. The percent signal change refers to the difference in BOLD response in relation to the global mean. y, MNI-coordinate in anterior-posterior direction.

In experiment 1, subjects performed tasks in which they had to attend either to the content (syllable task) or the level (loudness task) of speech sounds. Both tasks were one-back same/different judgments performed on sequences of syllables (see Experimental Procedures). Subjects attended to the same stimulus material for both tasks. If the modulation by attention is not specific, we would expect that the amplitude of the MGB activity will either be the same for the two tasks or greater for the more difficult task. We localized MGB, functionally, by contrasting blood oxygen level-dependent (BOLD) responses to speech sounds with responses to silence (Figure 1A and Figure S1 available online). In the group analysis (n = 16), only the response in the left MGB was significant. Within this region, we found that BOLD activity was greater for the syllable task than the loudness task (p < 0.05 family-wise error [fwe] corrected for multiple comparisons) (Figure 1B, Table S1, and Figure S2).

The results of experiment 1 indicate that the task-dependent modulation of MGB cannot be explained by nonspecific attention to the speech stimulus. Behaviorally, the syllable task was easier to do for subjects than the loudness task [Table S2; F(1,15) = 127.56, p < 0.001, n = 16]. Therefore, the syllable task should have required relatively less attention. However, we found the opposite effect; MGB activity in the syllable task was greater than in the loudness task.

In the second experiment, we tested a more specific hypothesis about the functional role of the task-dependent modulation of the MGB. Critical features for speech recognition vary rapidly over time at the rate of the phonemes (e.g., in the syllable “aba,” a transition from /a/ to /b/ to /a/) [19]. In contrast, speaker-relevant variables such as vocal-tract length or fundamental frequency are more stable over time (during normal conversation and also in our experiment). We hypothesized that adjusting receptive fields for fast-changing speech characteristics at the phoneme level is resolved at an early processing stage and requires more modulation of MGB than the temporally more stable speaker characteristics.

Subjects performed the same syllable task as in experiment 1. For the control task, however, subjects had to attend to the speaker characteristics (speaker task) of the same speech sounds. Within both functionally localized MGBs (Figure 1C and Figure S1), the syllable task elicits greater BOLD activity than the speaker task (p < 0.05 fwe corrected, n = 17) (Figure 1D, Table S1, and Figure S2). Note that behavioral performance was matched for the two tasks [Table S2; F(1,16) = 0.53, p = 0.48, n = 17].

The results are consistent with our hypothesis; the increase of responses in the MGB for the speech task, as compared to the speaker task, suggests that modulation of first-order thalamic responses is involved in processing fast time-varying features at the phoneme level.

**Figure 2:**
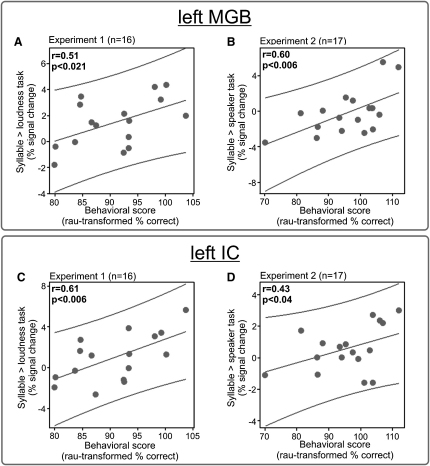
Correlation Analysis The plots show the positive correlation between the behavioral performance in the syllable task (as percent correct performance in rationalized arcsine units (rau) [40]) and the BOLD-signal change in the sensory auditory thalamus, the medial geniculate body (MGB) (A and B), and the auditory midbrain, the inferior colliculus (IC) (C and D), over subjects. The linear regression is shown with 95% individual prediction interval.

It is generally assumed that tuning of first-order thalamic receptive fields in nonhuman mammals leads to improved detection and discrimination of stimuli (although to our knowledge there is no experimental evidence for this assumption) [5]. Given this assumption, we would expect a positive correlation of behavioral performance with the amount of task-dependent modulation in human MGB during speech recognition. We found this to be the case for both MGBs, over both experiments (p < 0.05 fwe corrected, n = 33) (Figure S3). There were no significant differences in the strength of correlation between the two experiments (p = 0.6). Separate correlation analyses for the two experiments (Figures 2A and 2B and Table S3) revealed a positive correlation between performance and difference contrast, in experiment 1 for both MGBs. In experiment 2, the positive correlation was significant in the left MGB only.

Two previous fMRI studies have implicated thalamic structures in attention [20] or recognition success [21] during speech perception. However, it seems that activations were not located in sensory thalamic structures (i.e., the MGB), although this cannot be stated with certainty because of the specific acquisition and analysis techniques used in these studies. Activation maxima (in [20]: −13, −6, 12; in [21]: −0, −12, 5; Talairach coordinates) are more than 1.5 cm away from maxima reported for the MGB (e.g., in [22]: −15, −28, −5 [Talairach]; −15, −28, −8 [MNI]; see also Table S1). The distinction between primary sensory thalamus and other thalamic structures is important because only modulation of first-order nuclei, such as the MGB, indicates task-dependent modulation at an early sensory processing stage [8, 10].

Differences in brainstem evoked potentials between groups with relatively high or low language skills have been attributed to the inferior colliculus (IC) [23, 24]. Accordingly, we also examined the BOLD-responses of the ICs in the current experiments (Figure S4). They were not more active for the syllable task than the control tasks (i.e., loudness task [experiment 1] or speaker task [experiment 2]). However, there was a positive correlation of behavioral performance with the activation difference between the syllable and control tasks, in both ICs over both experiments (p < 0.05 fwe corrected, n = 33). Further analyses revealed a significant positive correlation for experiment 1 in both ICs, and in experiment 2, in the left IC (Figures 2C and 2D and Table S4). This correlation of task-dependent modulation with recognition success further suggests a possible role for IC in subjects with high language skills [23, 24].

In summary our findings show (1) that responses in auditory, first-order thalamus during the processing of speech sounds are task dependent, (2) that the response is strongest when processing features of speech sounds at the phoneme level, and (3) that the relative amplitude of the task-dependent modulation is correlated with individual behavioral performance. This suggests that a task-dependent, behaviorally relevant feedback mechanism supports the recognition of speech sounds at the level of sensory thalamus. In light of the massive corticothalamic connections, and their known influence on the response properties of thalamic neurons in animals [1–5, 13, 14], we speculate that the task-dependent modulation seen in the current experiments is a result of feedback from cortical areas. Such feedback could be direct (e.g., from auditory primary or association cortices) or indirect (i.e., via other structures such as the reticular nucleus) [25]. Although not the most parsimonious explanation, task-dependent modulation could also be mediated via the IC, which receives afferents from cortex and is one of the main input structures to the MGB [25, 26].

What kind of mechanism can account for these results? Experimental and theoretical accounts of brain function [27–29] emphasize the importance of an anatomical hierarchy that is organized according to the timescale of complex stimuli, in the natural environment. In brief, it is assumed that levels closer to the sensory input encode faster dynamics of the stimulus than levels further away from the sensory input. In accordance with this view, the MGB (as well as visual first-order thalamus [LGN] [30]) are tuned to high frequencies of temporal modulation (ca. 16 Hz in human MGB [27]) in relation to their associated primary sensory cortical areas [27, 29, 31]. In addition, in nonhuman animals, thalamic receptive fields change dynamically in response to inputs from cortical areas [4, 5]. For humans, the optimized encoding of relatively fast dynamics, e.g., at the phoneme level, is critical for speech recognition and communication [32–34]. Here, we suggest that slower dynamics encoded by auditory cortical areas [27, 29] provide predictions about input arriving at lower levels of the temporal-anatomic hierarchy [28]. In this view, these dynamic predictions modulate the response properties of the first-order sensory thalamus to optimize the early stages of speech recognition.

## Experimental Procedures

This section provides only essential information about the Experimental Procedures. For a more detailed description, see Supplemental Experimental Procedures.

### Participants

Sixteen subjects were included in experiment 1 (all right handed; eight female, eight male; aged 19–40 years; mean age of 26). Seventeen subjects were included in experiment 2 (all right handed; 6 female, 11 male; aged 20–37; mean age of 26).

### Stimuli

Stimuli were based on syllables recorded from a single speaker (16-bit resolution, 48 -kHz sample rate) and preprocessed with level balancing and perceptual centering as described previously [35]. Experiment 1 contained 96 syllables (48 consonant-vowel, 48 vowel-consonant). Experiment 2 contained 150 vowel-consonant-vowel syllables. We used vowel-consonant-vowel syllables in experiment 2 because preliminary behavioral studies indicated that they impart the same level of task difficulty for the syllable and speaker task. Stimulus versions with different speaker characteristics were synthesized from the recorded speech sounds with the STRAIGHT software package [36, 37].

### Experimental Design

For both experiments spoken syllables were concatenated to form syllable sequences (Supplemental Experimental Procedures—Example Stimuli). Before each sequence, participants received a visual instruction to either perform the syllable task or the control task (i.e., loudness task in experiment 1 or speaker task in experiment 2).

Both experiments included a second factor, which was the synthetic manipulation of voice characteristics. These manipulations were not of immediate interest to the main hypotheses of the current report and the results are therefore presented in the Supplemental Experimental Procedures.

#### Experiment 1

In experiment 1, all syllable-sequences lasted 9.44 s and contained eight syllable events (680 ms stimulus, 500 ms pause). Within each sequence there were three different syllables (e.g., /ga/, /ke/, and /la/; /mu/, /mi/, and /ka/; etc.) and three different values of sound level (values differed by 9–12 dB SPL). Syllable and sound level values were randomly presented with restrictions on the minimum and maximum number of changes within a sequence. Changes in syllable and sound level were independent of each other. In the speech task, subjects indicated via button press whether the current syllable was different from the previous one. In the loudness task, subjects indicated via button press whether the level of the current syllable was different from the previous one. During half of the syllable sequences, the vocal-tract length (VTL) of the speaker varied within the syllable sequence (VTL varies); during the other half, the VTL of the speaker was fixed (VTL same). Thus, we analyzed four experimental conditions: (1) syllable task, VTL varies; (2) syllable task, VTL same; (3) loudness task, VTL varies; and (4) loudness task, VTL same. The VTL values ranged from 10.6 to 21.7 cm.

#### Experiment 2

In experiment 2, all syllable-sequences lasted 8.4 s and contained six syllable events (1100 ms stimulus, 300 ms pause). Within each sequence there were three different syllables (e.g., /aga/, /ake/, and /ala/; or /esi/, /elu/, and /ero/; etc.) and three different speakers (i.e., different VTLs or different fundamental frequencies, see below). Again, the minimum and maximum numbers of changes were restricted, and variations in syllable and speaker were independent. In the speech task, subjects indicated via button press whether the current syllable was different from the previous one. In the speaker task, subjects indicated via button press whether the current speaker was different from the previous one. Half of the syllable sequences were spoken by speakers that differed in VTL but that had the same fundamental frequency, i.e., the same glottal pulse rate (GPR). In the remaining sequences, the speakers differed in GPR but not in VTL. Thus, the experiment had four experimental conditions: (1) syllable task, VTL varies; (2) syllable task, GPR varies; (3) speaker task, VTL varies; and (4) speaker task, GPR varies. The smallest values for the speaker manipulations were 95 Hz (GPR)/9.1 cm (VTL), and the largest values were 220 Hz (GPR)/20.3 cm (VTL). The values were chosen to simulate a speaker change, rather than a change in the voice quality of one speaker.

Both experiments also included silence conditions, which were 9.44 s in experiment 1 and 8.4 s in experiment 2. These conditions were used to locate the regions of interest (see below).

In both experiments, the order of sequences was randomized. Each sequence (with a specific stimulus combination) always occurred twice, once in the syllable task, and once in the control task.

### Scanning Procedure

The stimuli were delivered with a custom electrostatic system at 70 dB SPL.

After each syllable sequence, functional gradient-echo planar images (EPI) were acquired (sparse imaging [38, 39]) on a 3 Tesla-scanner (Siemens Allegra, Erlangen, Germany; 42 slices; −5 degree tilt; slice thickness 2 mm, interslice distance 1 mm; cardiac triggering).

### Data Analysis

Imaging data were analyzed with statistical parametric mapping implemented in SPM5 software (http//:www.fil.ion.ucl.ac.uk/spm) (see Supplemental Experimental Procedures). Population-level inferences about BOLD signal changes between conditions of interest were based on a random effects model that estimated the second level t statistic at each voxel.

#### Definition of Regions of Interest for the Categorical Analysis

For both experiments, we located the MGB and IC by the contrast all speech conditions > silence at the second level. For the regions of interest (ROI) definition, we used the MarsBaR toolbox (http://marsbar.sourceforge.net). The ROIs are displayed in Figure S1 (MGB) and Figure S4 (IC). The statistical maxima for the ROIs are listed in Table S1 (MGB) and Table S4 (IC).

#### Categorical Analysis

In the categorical analysis, the contrasts of interest were syllable task > loudness task (experiment 1) and syllable task > speaker task (experiment 2). Effects were considered significant if (1) the statistic maximum of the cluster was within the functionally defined ROI and (2) this was significant at p < 0.05, fwe corrected for multiple comparisons within the functionally defined ROI.

#### Definition of ROIs for the Correlation Analysis

The ROIs for the correlation analysis were defined as described above, except that the functional cluster was not derived from the contrast all speech conditions > silence, but from the functionally more specific contrast syllable task > loudness task (experiment 1) or syllable > speaker task (experiment 2) (MGB, Figure S1 and Table S1). Because there were no significant responses for this contrast in the IC, we used the same ROIs as for the categorical analysis (Figure S4).

#### Correlation Analysis

In the correlation analysis, the fMRI difference signal, between syllable and control tasks (i.e., loudness or speaker task) in MGB and IC, was correlated with the behavioral performance (in rationalized arcsine units [40]) in the syllable task. To estimate Pearson's statistics, we extracted the parameter estimates from the region of interest at the voxel where we found the maximum value of the statistic. These values and the behavioral scores were then analyzed with SPSS 12.02. To estimate differences in correlation between the two experiments, we used a univariate ANOVA.

#### Analysis of Behavioral Data

The behavioral data were analyzed with a repeated-measures ANOVA and post hoc paired t tests in SPSS 12.02 (Table S2). The statistical results are described in the main text and in the legend of Table S2.

## Supplemental Data

Document S1. Supplemental Experimental Procedures, Five Figures, and Four Tables

Audio S1. Exp1_VTL_varies_v

Audio S2. Exp1_VTL_varies_w

Audio S3. Exp1_VTL_fixed_v

Audio S4. Exp1_VTL_fixed_w

Audio S5. Exp2_VTL_varies

Audio S6. Exp2_GPR_varies
