# Supplementary material for: Task-Dependent Modulation of Medial Geniculate Body Is Behaviorally Relevant for Speech Recognition
Source: Curr Biol. 2008 Dec 9;18(23-2):1855–9. doi: 10.1016/j.cub.2008.10.052 (PMC2631608; doi:10.1016/j.cub.2008.10.052)
Supplement: Document S1. Supplemental Experimental Procedures, Five Figures, and Four Tables [file mmc1.pdf]

# Supplemental Data

# Task-Dependent Modulation of Medial Geniculate Body Is Behaviorally Relevant for Speech Recognition

**Katharina von Kriegstein, Roy D. Patterson, and T.D. Griffiths**

## Supplemental Experimental Procedures

### Participants

Four additional subjects were not included in the analyses because of excessive movement during scanning (2 subjects, Experiment 1) or lack of equal performance in the syllable and speaker task (2 subjects, Experiment 2). All subjects gave informed consent, and the experiment was carried out with the approval of the Institute of Neurology Ethics Committee, London. None of the subjects had any history of audiological or neurological disorder, and all had normal structural MRI brain scans.

### Experimental Design and Stimuli

Prior to the scanning, subjects were briefly familiarized with the tasks and type of stimuli in a practice session. In both experiments, the session comprised 8 syllable sequences (4 for the syllable task and 4 for the control task, i.e. loudness task/Experiment 1 or speaker task/Experiment 2).

<u>Experiment 1</u>

In Experiment 1, syllable and sound level values were randomly presented within the sequence, with two restrictions: (i) each sound-level value/syllable occurred at least twice and (ii) changes between two consecutive sound-level values/syllables occurred between three and five times within one sequence.

In the conditions with varying VTL (VTL varies), subjects listened to sequences of syllables within which VTL varied randomly; in the conditions with fixed VTL (VTL same), the value was randomly chosen from within the

VTL range, and remained the same throughout that sequence. In total there were eight values of VTL, equally spaced in logarithmic terms (21.7, 18.5, 16.9, 15.4, 14.1, 12.8, 11.7, 10.6 cm). VTL is roughly 10% of body height in humans, so the VTL values correspond to speakers with heights ranging from about 1.1 m to 2.2 m. The range of VTL exceeds the normally occurring range at the upper limit (i.e. a body size of 2.2 m is very unusual). Previous behavioural studies have shown that speech recognition as well as the judgment of speaker characteristics is robust to even this extreme case of VTL manipulation. The same holds for unusual combinations of GPR and VTL (such as a child of 1.1 m with an GPR of 160 Hz) [1].

Besides the experimental factors described in the main text, Experiment 1 also contained a third factor, which was intra-speaker voice quality. Specifically half of the syllable sequences were voiced, the other half whispered. The GPR for the voiced sounds was fixed at 160 Hz. The whispered sounds were produced by resynthesizing with a broadband noise carrier, and lifting the spectrum 6-dB per octave to match the spectral slope of whispered speech [2]. The sound levels were chosen to match the loudness discriminability for voiced and whispered stimuli, as indicated by informal listening. The voiced syllables were presented at 49, 61, and 73 dB RMS SPL; the whispered syllables were presented at 53, 63, and 72 dB RMS SPL. The voicing factor was included because the experiment is part of a larger research programme concerned with speech perception in the context of different intra- and inter-speaker characteristics, the results of which will be reported elsewhere. Voiced syllables were recognized equally well as whispered syllables (voiced: 87.56% (SE1.36); whispered: 88.63% (SE1.16)). After checking that there were no interactions between task and voice quality, and that the simple main effects (syllable-task/voiced > loudness-task/voiced; syllable-task/whispered > loudness-task/whispered) were of similar magnitude within our regions of interest (i.e. MGB and IC), we collapsed the analysis across the voicing variable. Accordingly, there were the four experimental conditions described in the main text: (*i*) syllable-task, different VTL; (*ii*) syllable-task, same VTL; (*iii*) loudness-task, different VTL; (*vi*) loudness-task, same VTL. There were 64 sequences/condition in the experiment (i.e., 32 with voiced syllables and 32 with whispered syllables).

Experiment 2

In Experiment 2, syllables and speakers were randomly presented within the sequence with two restrictions: (i) each syllable/speaker occurred at least twice and (ii) changes between two consecutive syllables/speakers occurred between two and three times within a sequence.

In the conditions with varying VTL (VTL varies), VTL varied randomly and the GPR was fixed throughout the sequence; in the conditions with fixed VTL, GPR varied randomly throughout the sequence (GPR varies). In total there were three VTL values (9.1, 13.6, 20.3 cm) and three GPR values (95, 147, and 220 Hz). These VTL values correspond to speakers with heights of approximately 0.9 m, 1.4 m and 2.0 m, respectively. The GPR and VTL values were chosen because preliminary behavioural studies indicated that subjects in general perceive these values as a change of speaker rather than a change of the voice characteristics of one speaker. In the speaker task, subjects were asked to only score two consecutive syllable events as different if they clearly perceived a change of speaker rather than a change of the voice of one speaker.

There were 40 sequences for each of the four conditions in the experiment (*i*) syllable-task, VTL differs; (*ii*) syllable-task, GPR differs; (*iii*) speaker-task, VTL differs; (*vi*) speaker-task, GPR differs.

Resynthesizing stimuli from one recorded speaker to simulate different speakers prevents influences of time-varying speaker idiosyncrasies [3,4], which could occur in recordings taken from different natural speakers. Time-varying speaker idiosyncrasies (e.g. articulation habits, [3]) can potentially be used as additional cues for speaker recognition. This would have been a confound especially in Experiment 2, because the difference between recognition of fast time-varying speech and more stable speaker cues is at the heart of our hypothesis.

## Scanning Procedure

Cardiac triggering was applied to lessen artefacts caused by pulsatile motion of the brainstem. Because of this, there was a variable scan repetition time

(TR (time to repeat): 2.73s+length of stimulus presentation + time to next pulse; TE (time to echo): 65ms). The 42 transverse slices of each brain volume covered the entire brain. The task instruction was presented during the last ten slice acquisitions of each volume. It was followed by a fixation cross displayed during the subsequent stimulus sequence. Experiment 1 included 222 brain volumes for each subject (3 runs of 74 volumes each). Experiment 2 included 210 brain volumes for each subject (5 runs of 42 volumes each). Subjects were allowed to rest for several minutes between runs. The first two volumes were discarded from each run.

**Data analysis**

Scans were realigned, unwarped and spatially normalized [5] to MNI standard stereotactic space [6] and spatially smoothed with an isotropic Gaussian kernel of 4 mm full-width-at-half-maximum (FWHM). We also performed a second analysis with a larger smoothing kernel (8mm FWHM) to investigate the cortical activation of the contrast of interest (syllable task > loudness task, syllable task > speaker task; **Figure S5**). For all analyses, statistical parametric maps were generated by modelling the evoked hemodynamic response for the different stimuli as boxcar functions convolved with a synthetic hemodynamic response function in the context of the general linear model [7]. To test for effects of hemisphere, we modelled the experimental contrasts of interest in an analysis in which we concatenated the normal and right-left flipped functional images of each single subject in a first level single-subject design matrix.

<u>Categorical analysis</u>

*Definition of Region of Interests (ROI)*

For both experiments we located the MGB and IC by the contrast all speech conditions > silence at the second level. Responses in these regions were considered significant at $p<0.001$, uncorrected. In Experiment 1, MGB was significantly activated in the left hemisphere; in Experiment 2, significant activity was bilateral (**Table S1**). The effect in right MGB in Experiment 1 was below the significance threshold ($p=0.02$ uncorrected for multiple comparisons). Inferior colliculus (IC) was activated, bilaterally, in both

experiments (**Table S4**). ROIs were defined by the functional cluster for the contrast all speech conditions > silence in combination with a sphere centred at the location of the maximum statistic.

*Contrasts of interest*

In both experiments the contrast of interest was syllable task > control task. In Experiment 1 the contrast syllable > loudness task corresponds to: ((syllable task/VTL varies) + (syllable task/VTL same)) – ((loudness task/VTL varies) + (loudness task/VTL same)). In Experiment 2 the contrast syllable vs. speaker task corresponds to: ((syllable task/VTL varies) + (syllable task/GPR varies)) – ((speaker task/VTL varies) + (speaker task/GPR varies)).

For the plots in **Figure 1** and **Figure S2,** parameter estimates were extracted for each condition separately from the voxel, at which we found the maximum statistic for the group for the contrast of interest (syllable > control task). These values were then entered into a repeated measures ANOVA with the factors task (syllable, control) and stimulus (Experiment 1: VTL varies, VTL same; Experiment 2: VTL varies, GPR varies) and plotted using SPSS 12.02 (SPSS Inc, Chicago, IL, USA). The plotted values correspond to percent signal change relative to the global mean.

<u>Correlation analysis</u>

We performed two correlation analyses. We tested with the original syllable percent-correct scores, as well as with percent-correct scores transformed to rationalized arcsine units (rau) [8]. The latter was done to avoid the compressive effects of the percent correct scale, which occurs when a substantial proportion of the data points are above 80% [8]. The results reported are from the analysis with the scores in rationalized arcsine units. Between the two analyses, there were some quantitative but no qualitative differences in the MGB results. However, in left IC in Experiment 2, the BOLD responses are positively correlated with the behavioral score only when the rau transform was used.

Test for interactions between hemisphere and task

For the categorical as well as the correlation analysis, we also tested for interactions between task and hemisphere. This was motivated by the asymmetric sampling theory for speech processing [9]. Specifically we tested whether there is a differential preference for processing time-varying and time-constant speech features in left and right MGB respectively. The results do not suggest that there is a clear difference. Although there was a task by hemisphere interaction ((syllable task/left MGB - loudness task/left MGB) – (syllable task/right MGB - loudness task/right MGB)) in Experiment 1 (MNI-coordinates: -10, -24, -10, Z=3.72), there was no similar interaction in Experiment 2. Furthermore, the interaction in Experiment 1 has to be interpreted with caution because there was no significant activation in the right MGB for all speech conditions > silence, so we cannot exclude the involvement of a difference in sensitivity. It is interesting to note that in Experiment 2, positive correlation between the difference contrast (syllable > speaker task) and the behavioural score was significant only in the left hemisphere (i.e. in left MGB and left IC). There was an interaction between positive correlation and hemisphere in MGB and IC, indicating that activity in left MGB correlated to a greater extent with the behavioural score (in rau, [8]) than activity in right MGB (MGB: -18, -20, -10, Z=1.73; IC -6, -32, -14, Z=2.06).

## EXAMPLE STIMULI

Examples for the syllable sequences used in the two experiments are available as supplementary material.

Experiment 1

Each file contains a sequence of 8 syllable events (680ms, 500 ms pause). Task instructions (either 'syllable task' or 'loudness task') were given visually before each sequence.

VTL varies: 'Exp1_VTL_varies_v.wav'; 'Exp1_VTL_varies_w.wav'

VTL same: Exp1_VTL_fixed_v.wav'; 'Exp1_VTL_fixed_w.wav'

(v, voiced; w, whispered)

<u>Experiment 2</u>

Each file contains a sequence of 6 syllable events (1100ms, 300 ms pause). Task instructions (either ‘syllable task’ or ‘speaker task’) were given visually before each sequence.

VTL varies: ‘Exp2_VTL_varies.wav’

GPR varies: ‘Exp2_GPR_varies.wav’

**Supplemental References**


1. Smith DRR, Patterson RD, Turner R, Kawahara H, Irino T: **The processing and perception of size information in speech sounds.** *J Acoust Soc Am* 2005, **117:** 305-318.

2. Fujimura O, Lindqvist J: **Sweep-tone measurements of vocal-tract characteristics.** *J Acoust Soc Am* 1971, **49:** 541-558.

3. Abercrombie D. Elements of General Phonetics. 1967. Edinburgh, Edinburgh University Press.

4. Garvin PL, Ladefoged P: **Speaker Identification and Message Identification in Speech Recognition.** *Phonetica* 1963, **9:** 193-199.

5. Friston KJ, Ashburner J, Frith CD, Poline JB, Heather JD, Frackowiak RSJ: **Spatial registration and normalisation of images.** *Human Brain Mapping* 1995, **2:** 165-189.

6. Evans AC, Collins DL, Mills SR, Brown ED, Kelly RL, Phinney RE: **3D statistical neuroanatomical models from 305 MRI volumes.** *Proc IEEE Nucl Sci Symp Med Imag Conf* 1993, **3:** 1813-1817.

7. Friston KJ, Holmes AP, Worsley KJ, Poline JP, Frith CD, Frackowiak RSJ: **Statistical parametric maps in functional imaging: A general linear approach.** *Hum Brain Mapp* 1995, **2:** 189-210.

8. Studebaker GA: **A "rationalized" arcsine transform.** *J Speech Hear Res* 1985, **28:** 455-462.

9. Poeppel D: **The analysis of speech in different temporal integration windows: cerebral lateralization as 'asymmetric sampling in time'.** *Speech Communication* 2003, **41:** 245-255.

10. Irino T, Patterson RD: **Segregating information about the size and shape of the vocal tract using a time-domain auditory model: The stabilised wavelet-Mellin transform.** *Speech Communication* 2002, **36:** 181-203.

11. von Kriegstein K, Warren JD, Ives DT, Patterson RD, Griffiths TD: **Processing the acoustic effect of size in speech sounds.** *Neuroimage* 2006, **32:** 368-375.

12. von Kriegstein K, Eger E, Kleinschmidt A, Giraud AL: **Modulation of neural responses to speech by directing attention to voices or verbal content.** *Brain Res Cogn Brain Res* 2003, **17:** 48-55.

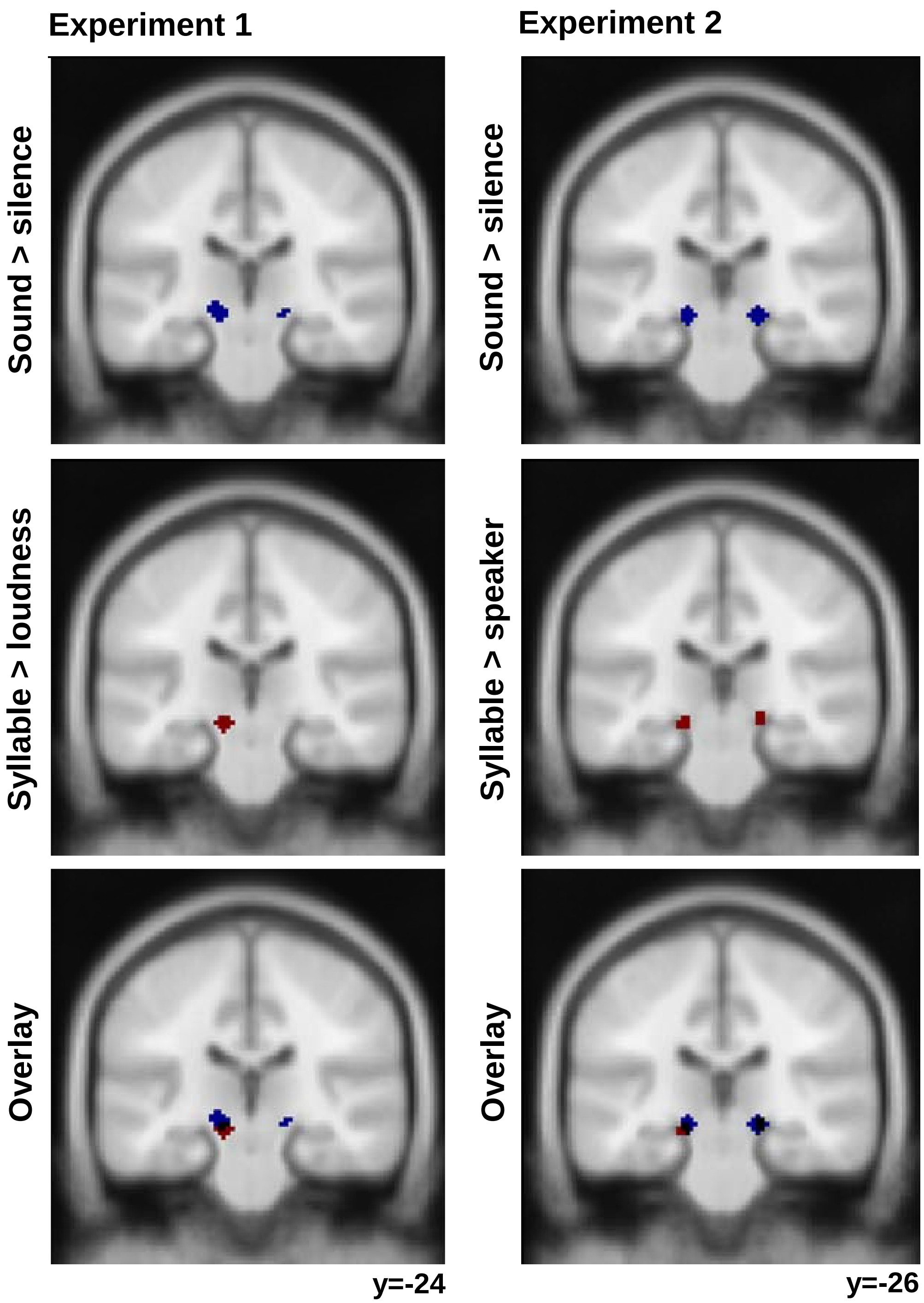


**Figure S1** Regions of interest for the MGB in both experiments, displayed on coronal slices in MNI-space at y=-24 mm (Experiment 1), and y=-26 (Experiment 2). 'Sound > silence' refers to the contrast 'all speech conditions > silence.' 'Syllable > loudness' refers to the contrast 'syllable task > loudness task' in Experiment 1; 'Syllable > speaker' refers to the contrast 'syllable task > speaker task' in Experiment 2.

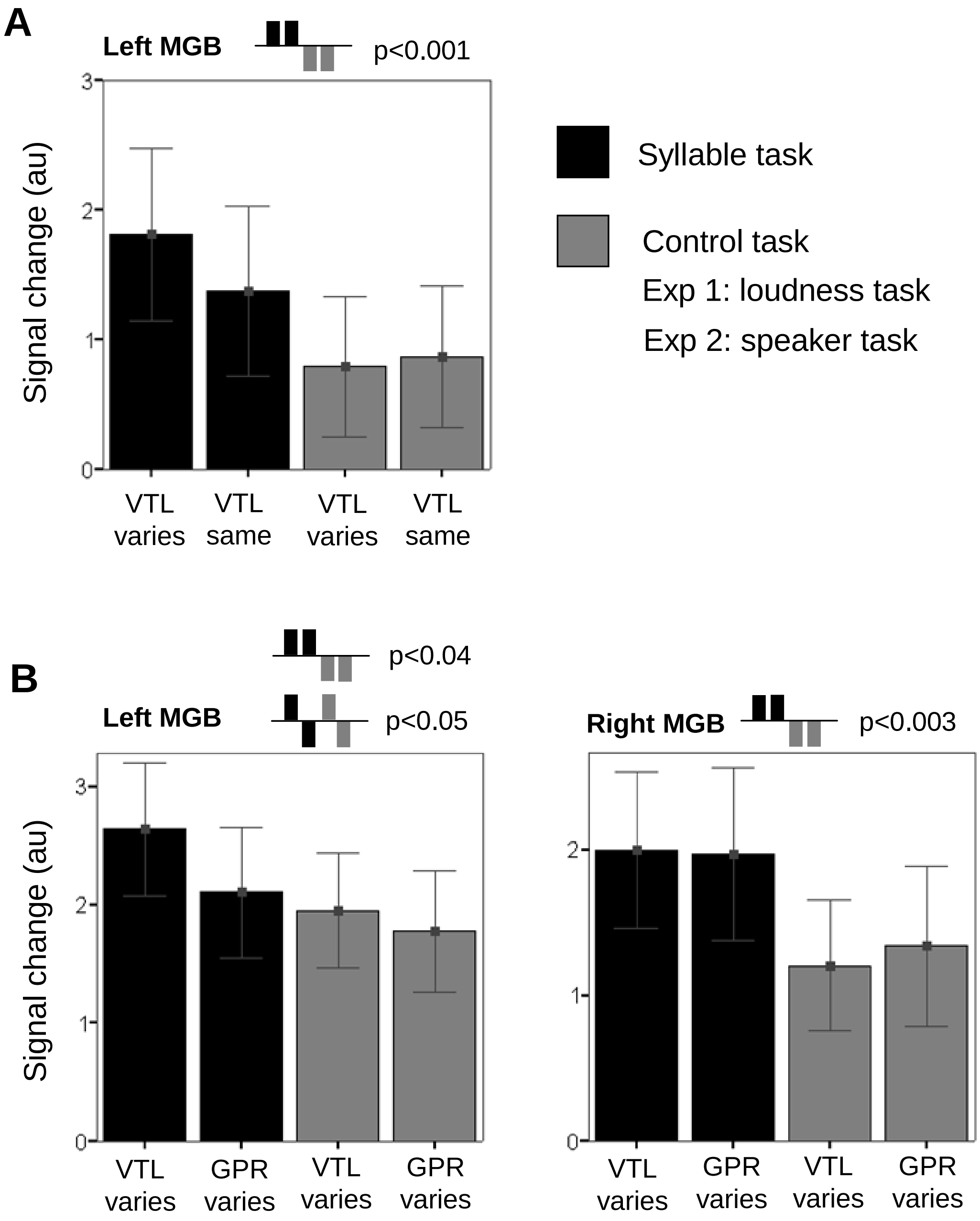


**Figure S2** Plot of parameter estimates extracted (**A**) from left MGB in Experiment 1 (n=16) and (**B**) from both MGBs in Experiment 2 (n=17), for each experimental condition separately. The parameter estimates for each experimental condition are contrasted against the silent baseline. Besides the task factor, both experiments included a second factor, which was the synthetic manipulation of speaker characteristics. Specifically, within the experimental sequences of Experiment 1 syllables were either spoken by speakers with different vocal tract length (VTL varies) or by a speaker with the same vocal tract length (VTL same). In Experiment 2, syllables could either be spoken by speakers with different vocal tract length (VTL varies) or by speakers with different glottal pulse rate (GPR varies) (see Supplemental Experimental Procedures). The acoustic effect of vocal tract length (VTL) in speech sounds is reflected in the spectrum (or timbre) of the sound [10]. The manipulation of VTL adds to the change in spectro-temporal complexity between the

consecutive syllables, but not within syllables. There was no significant interaction between the syllable task and the VTL manipulation in the categorical analysis. The significant main effects are indicated at the top of the individual plots. The main effect of VTL in left MGB in Experiment 2 is consistent with a similar effect in a previous report [11]. Error bars represent the Mean +/- 1.0 SE. VTL, vocal tract length; GPR, glottal pulse rate; % signal change refers to the difference in BOLD response in relation to the global mean.

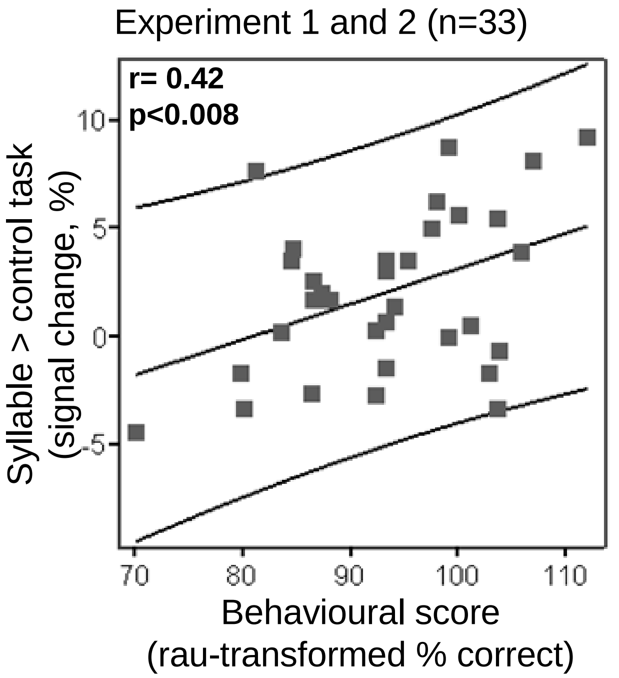


**Figure S3** Correlation analysis over both experiments. The plot shows the positive correlation between the behavioural performance in the syllable task (as percent correct performance in rationalized arcsine units, [8]) and the BOLD-signal change (for the contrast ‘syllable > control task’) in MGB over subjects and over experiments. Control task refers to ‘syllable task > loudness task’ in Experiment 1 and ‘syllable task > speaker task’ in Experiment 2. % signal change refers to the difference in BOLD response in relation to the global mean. The linear regression is shown with 95% individual prediction interval.

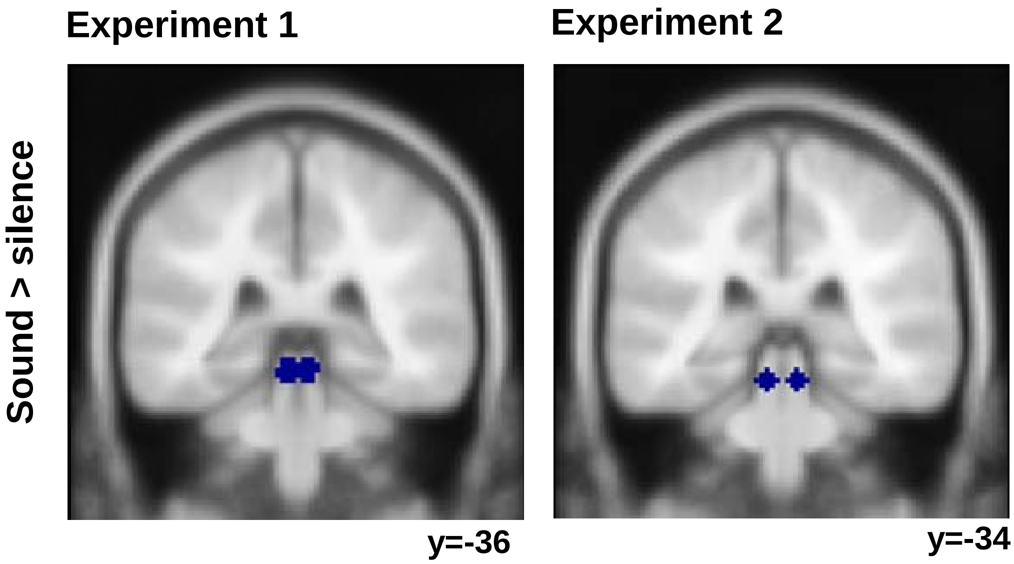


**Figure S4** Regions of interest for the inferior colliculus (IC) in both experiments as defined by the contrast all speech conditions > silence.

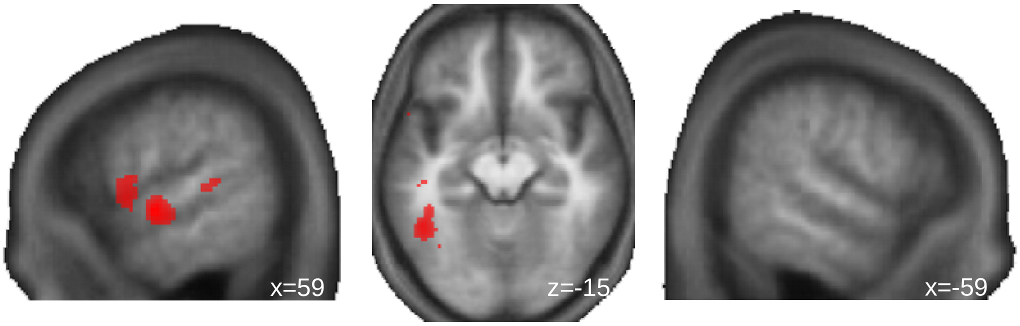


**Figure S5** Cortical activation for the categorical analysis. The statistical parametric map shows a conjunction analysis for the two Experiments for the syllable task > control task (Experiment 1: syllable > loudness task; Experiment 2: syllable > speaker task) overlaid on the mean structural image of both groups, $p<0.001$ uncorrected. The results are in accordance with findings for a similar contrast in a previous study [12].

| Experiment 1 (n=16) | x | y | z | Z | Experiment 2 (n=17) | x | y | z | Z |
|---|---|---|---|---|---|---|---|---|---|
| **both MGB** | | | | | **both MGB** | | | | |
| sound > silence | -/+14 | -24 | -8 | 3.02 | sound > silence | +/-16 | -26 | -8 | 5.01 |
| syllable > loudness | -/+14 | -26 | -10 | 2.35 | syllable > speaker | +/-16 | -24 | -10 | 2.72 |
| **left MGB** | | | | | **left MGB** | | | | |
| sound > silence | -14 | -24 | -6 | 3.06 | sound > silence | -16 | -26 | -8 | 4.21 |
| syllable > loudness | -12 | -24 | -10 | 3.34 | syllable > speaker | -18 | -24 | -10 | 1.98 |
| **right MGB** | | | | | **right MGB** | | | | |
| sound > silence | 18 | -24 | -6 | 1.80 | sound > silence | 16 | -26 | -8 | 4.83 |
| syllable > loudness | - | - | - | - | syllable > speaker | 16 | -24 | -8 | 2.88 |

**Table S1** Local activation maxima for the categorical analysis. ‘Syllable > loudness’ refers to the contrast ‘syllable task > loudness task’ in Experiment 1; ‘Syllable > speaker’ refers to the contrast ‘syllable task > speaker task’ in Experiment 2. x, y, z are the MNI coordinates of the local maxima (in millimeters). Z, standard score.

| Experiment 1 (n=16) | % correct | SE | Experiment 2 (n=17) | % correct | SE |
|---|---|---|---|---|---|
| **syllable task** | **88.09** | **1.23** | **syllable task** | **90.75** | **1.71** |
| VTL varies | 86.49 | 1.30 | VTL varies | 90.97 | 1.76 |
| VTL same | 89.69 | 1.24 | GPR varies | 90.52 | 1.72 |
| **loudness task** | **75.87** | **1.23** | **speaker task** | **89.03** | **2.30** |
| VTL varies | 74.27 | 1.21 | VTL varies | 90.76 | 2.18 |
| VTL same | 77.48 | 1.35 | GPR varies | 87.29 | 2.57 |

**Table S2** Behavioural Results. In Experiment 1 there was a main effect of task (Syllable vs. loudness task: F1,15=127.56, $p<0.001$) and stimulus (VTL varies vs VTL same: $F(1,15)=38.03$, $p<0.001$). There was no task x stimulus interaction. In Experiment 2 there was no main effect of task (Syllable vs speaker task: $F(1,16)=0.53$, $p=0.48$). There was a stimulus x task interaction ($F(1,16)=5.25$, $p=0.036$). Post-hoc t-tests showed that the interaction is due to differences in performance levels for the speaker tasks in conditions where GPR or VTL vary (varying GPR vs. varying VTL in speaker task: $t=2.79$, df=16, $p=0.013$). There were no further simple main effects (syllable vs speaker task when VTL varies ($p=0.93$); syllable vs speaker task when GPR varies ($p=0.23$); VTL varies vs GPR varies during syllable task ($p=0.53$). SE, standard error of the mean.

| | both MGB | | | | left MGB | | | | right MGB | | | |
|---|---|---|---|---|---|---|---|---|---|---|---|---|
| | x | y | z | Z | x | y | z | Z | x | y | z | Z |
| **Exp1&2 (n=33)** | +/-18 | -24 | -10 | 2.69 | -16 | -20 | -12 | 2.49 | 18 | -26 | -8 | 1.91 |
| **Exp 1 (n=16)** | +/-18 | -24 | -10 | 2.59 | -14 | -24 | -10 | 2.24 | 18 | -24 | -6 | 2.93 |
| **Exp 2 (n=17)** | - | - | - | - | -20 | -22 | -12 | 2.45 | - | - | - | - |

**Table S3** Local activation maxima in MGB for the correlation analysis of behavioural and fMRI data. The reported maxima show a significant positive correlation of the behavioural performance in the syllable task (as percent correct performance in rationalized arcsine units, [8]) with the amount of BOLD-signal change between conditions in MGB over subjects. x, y, z are the MNI coordinates of the local maxima (in millimetres). Z, standard score.

| Experiment 1 (n=16) | | | | | Experiment 2 (n=17) | | | | |
|---|---|---|---|---|---|---|---|---|---|
| | x | y | z | Z | | x | y | z | Z |
| **both IC** | | | | | **both IC** | | | | |
| sound > silence | +/-4 | -36 | -12 | 4.7 | sound > silence | +/-6 | -34 | -14 | 5.6 |
| syllable > loudness | - | - | - | - | syllable > speaker | - | - | - | - |
| positive correlation | +/-6 | -34 | -14 | 2.8 | positive correlation | - | - | - | - |
| **left IC** | | | | | **left IC** | | | | |
| sound > silence | -4 | -36 | -12 | 4.4 | sound > silence | -6 | -34 | -16 | 5.1 |
| syllable > loudness | - | - | - | - | syllable > speaker | - | - | - | - |
| positive correlation | -6 | -32 | -14 | 2.5 | positive correlation | -4 | -34 | -18 | 1.7 |
| **right IC** | | | | | **right IC** | | | | |
| sound > silence | 4 | -36 | -12 | 4.4 | sound > silence | 6 | -34 | -16 | 4.9 |
| syllable > loudness | - | - | - | - | syllable > speaker | - | - | - | - |
| positive correlation | 6 | -34 | -14 | 2.8 | positive correlation | - | - | - | - |

**Table S4** Local activation maxima for the categorical and correlation analyses in the Inferior Colliculus (IC). ‘Syllable > loudness’ refers to the contrast ‘syllable task > loudness task’ in Experiment 1; ‘Syllable > speaker’ refers to the contrast ‘syllable task > speaker task’ in Experiment 2. x, y, z are the MNI coordinates of the local maxima (in millimetres). Z, standard score.
